# Angiopoietin-2 is released during anaphylactic hypotension in anesthetized and unanesthetized rats

**DOI:** 10.1371/journal.pone.0242026

**Published:** 2020-11-17

**Authors:** Tao Zhang, Toshishige Shibamoto, Mamoru Tanida, Makoto Taniguchi, Yuhichi Kuda, Wei Yang, Yasutaka Kurata

**Affiliations:** 1 Department of Physiology II, Kanazawa Medical University, Ishikawa, Japan; 2 Department of Colorectal and Hernia Surgery, The Fourth Affiliated Hospital of China Medical University, Shenyang, China; 3 Department of Life Science, Medical Research Institute, Kanazawa Medical University, Ishikawa, Japan; 4 Department of Infectious Disease, Shengjing Hospital of China Medical University, Shenyang, China; Max Delbruck Centrum fur Molekulare Medizin Berlin Buch, GERMANY

## Abstract

Angiopoietin (Angpt)-2, a permeability-increasing growth factor, is involved in vascular leakage of sepsis and acute lung injury, and could be released from endothelium in response to anaphylaxis-related secretagogues such as histamine and leukotrienes, or cytokines. However, roles of Angpt-2 in the hyperpermeability during systemic anaphylaxis are not known. Thus, we determined plasma levels of Angpt-2 and cytokines and vascular permeability during anaphylactic hypotension in unanesthetized rats. Anaphylaxis was induced by an intravenous injection of ovalbumin antigen. Mean arterial blood pressure (MBP) was measured, and hematocrit (Hct) and plasma levels of Angpt-2 and cytokines were assessed for 24 h after antigen injection. Separately, vascular permeability was measured in various organs using the Evans blue dye method, and Angpt-2 mRNA expression in liver was measured. After antigen injection, MBP decreased to the nadir at 6 min, and returned to baseline at 45 min, and Hct peaked at 20 min and thereafter progressively declined, suggesting that vascular leak and hypotension occurred within 20 min. Plasma Angpt-2 levels began to increase significantly at 1 h after antigen, reaching the peak 2.7-fold baseline at 6 h with a return to baseline at 24 h. Detected cytokines of IL-1α, IL-1β, IL-6, IL-10, and TNF-α peaked 1 or 2 h after antigen. Angpt-2 mRNA increased at 2 h and showed an increasing tendency at 6 h. Vascular permeability in bronchus, trachea, intestines, mesentery and skeletal muscle was increased at 10 min but not at 6 h after antigen. In addition, we confirmed using anesthetized rat anaphylaxis models that plasma Angpt-2 levels increased at 1 h after antigen. In conclusion, plasma Angpt-2 is elevated presumably due to increased cytokines and enhanced gene transcription during anaphylaxis in anesthetized and unanesthetized rats.

## Introduction

Anaphylactic shock is a sudden, life-threatening allergic reaction associated with systemic hypotension [1]. An increase in vascular permeability is one of the most important pathogenic factors for anaphylactic shock, because it causes plasma extravasation, resulting in decreases in circulating blood volume and venous return, and finally systemic hypotension. Chemical mediators such as platelet-activating factor and histamine play crucial roles in the anaphylaxis-induced increase in vascular permeability: histamine increases vascular permeability not only by acting directly on vascular endothelial cell barrier of adherens junctions [2, 3] but also by generating the other edematogenic substance of nitric oxide (NO) [4]. The involvement of NO in the increased permeability during systemic anaphylaxis is well established [5, 6]. Another candidate for the edematogenic factor associated with anaphylaxis is angiopoietin-2 (Angpt-2), a permeability-increasing endothelial growth factor. Indeed, Angpt-2 is reported to be involved in the vascular leakage disorders of sepsis [7] and acute lung injury [8]. Angpt-2 is released from the Weibel-Palade body in the vascular endothelium, which is induced in vitro by anaphylactic mediators of histamine [9], leukotrienes [10] and serotonin [11]. Particularly, the histamine-induced increase in vascular permeability was attenuated in Angpt-2-deficient mice [12]. However, roles of Angpt-2 in the hyperpermeability during systemic anaphylaxis are not known. On the other hand, it is reported that some cytokines such as TNF-α and IL-6 contribute to Angpt-2 production [9, 13, 14]. Thus, we determined the plasma levels of Angpt-2 and cytokines, and vascular permeability of various organs during anaphylactic hypotension in unanesthetized rats. In addition, Angpt-2 mRNA expression was also measured.

## Materials and methods

### Animals

Sixty-six male Sprague-Dawley rats weighing 370±8 g (Japan SLC, Shizuoka, Japan) were maintained at 23°C and under pathogen-free conditions on a 12:12-h dark/light cycle and allowed food and water ad libitum. The present experiments were conducted according to the National Institutes of Health guide for the care and use of Laboratory animals (NIH Publications No. 8023, revised in 1978) and approved by the Animal Research Committee of Kanazawa Medical University (2016–51). Rats were divided into two groups of the shock and control groups: the shock group animals were sensitized with subcutaneous injections of ovalbumin antigen (1 mg, 0.5 ml) and complete Freund’s adjuvant (0.5 ml), as described previously [15, 16], while the control group animals were injected with adjuvant only without ovalbumin. Two weeks after injection, rats were used for the following experiments.

All drugs were purchased from Sigma Chemical Company (St Louis, MO, USA) and were dissolved in saline. The lot number of the ovalbumin was SLBQ9036V.

### Unanesthetized rat experiment

#### Surgery

Ovalbumin-sensitized (n = 6) and non-sensitized (n = 6) rats were anesthetized by intraperitoneal injections of medetomidine hydrochloride (0.375 mg/kg), midazolam (2 mg/kg), and butorphanol tartrate (5 mg/kg), and placed supinely on a heating pad. The left femoral artery and vein were catheterized with polyethylene tubings (ID 0.5 mm and OD 0.8 mm) for measurement of mean arterial blood pressure (MBP) and injections of the antigen, saline and drugs, respectively. The tubings were subcutaneously transfixed and their ends were fixed at the neck. Following surgery, rats were placed into their home cages with a heating pad and monitored until recovery.

#### Protocol

Following recovery for at least 24 h after the surgery, the rats were placed in a custom-made plastic cage (LxWxH, 25x21x22 cm) with the exteriorized catheters connected to a cannula swivel (model TCS2-21, Tsumura; Tokyo, Japan) for free-moving, as reported previously [17]. MBP was digitally recorded at 40 Hz by PowerLab. At baseline, blood (0.3 ml) was sampled from the femoral artery for measurement of plasma concentrations of Angpt-2 and cytokines, and hematocrit (Hct), followed by an intravenous injection with the same volume of saline for replacement. Then, 0.6 mg ovalbumin in 300 μl saline was intravenously injected. At 6, 20, and 60 min, and 2, 6 and 24 h after antigen injection, blood (0.3 ml) was sequentially sampled. After MBP measurement for 1 h, the rats were free from the swivel system. At 24 h after antigen, the rats were euthanized with pentobarbital (125 mg/kg, i.v.).

#### Plasma Angpt-2 concentration measurement

Blood samples were transferred immediately to chilled tubes containing EDTA and centrifuged (1200×g, 10 min, 4°C). The plasma samples were separated and divided into two plastic tubes for measurement of Angpt-2 and cytokines, and stored at -80°C. Plasma Angpt-2 levels were assessed using the Mouse/Rat Angiopoietin-2 Quantikine ELISA Kit (R&D Systems, Minneapolis, USA) according to manufacturer’s guidelines. Samples were diluted and incubated at room temperature for 2 h. Incubation and wash cycles were repeated with the addition of conjugate and substrate solution. Finally, stop solution was added, and intensity was read using a microplate reader (Infinite F50, Tecan Japan, Kanagawa, Japan).

#### Plasma cytokine concentration measurement

The assays of IL-1α, IL-1β, IL-2, IL-4, IL-5, IL-6, IL-10, IL-12, IL-13, GM-CSF, TNF-α, and IFN-γ in the plasma sample were performed by using a Bio-Plex Rat Cytokine Th1/Th2 12Plex kit (1×96-well) (Bio-Rad Laboratories, Hercules, CA, USA), and the Bio-Plex 200 system and the Bio-Plex Manager software (Bio-Rad Laboratories) according to the manufacturer's instruction. Plasma was diluted 1:4 for all assays. The limits of detection for each cytokine were as follows: IL-1α (1.9 pg/mL), IL-1β (4.7 pg/mL), IL-2 (30.7 pg/mL), IL-4 (0.9 pg/mL), IL-5 (11.7 pg/mL), IL-6 (19.5 pg/mL), IL-10 (2.6 pg/mL), IL-12 (12.4 pg/mL), IL-13 (12.7 pg/mL), GM-CSF (1.2 pg/mL), TNF-α (34.7 pg/mL), and IFN-γ (3.3 pg/mL). Each sample was tested once.

#### Vascular permeability measurement

Separately from the Angpt-2 measurement experiments, the vascular permeability was measured by quantifying the Evans blue dye (EB; Sigma-Aldrich, ON, Canada) bound to albumin in various tissues such as the trachea, bronchus, mesentery, small intestine, and limb skeletal muscle. Rats either sensitized (n = 10) or non-sensitized (n = 10) with the antigen ovalbumin were instrumented in the same manner as the Angpt-2 measurement experiments, as described above. Rats were intravenously injected with EB (27 mg/kg, 1%) just before antigen injection or 6 h after antigen. At 10 min after EB injection, the animals were euthanized with pentobarbital and perfused with saline through the left ventricle at constant pressure of 100 mmHg over 5 min to remove intravascular EB. The tissues were removed, weighed, and stored in the refrigerator (-80°C) for later measurement. The tissue sample was immersed in formamide (1 mL for trachea and bronchus; 5 mL for the others) and incubated at 60°C for 48 h. Then, the formamide solution was transferred to a clear 96-well plate (160 μL per well). The optical density of the supernatant was determined using the microplate reader at 620 nm. The concentration of EB in the tissue was determined from the generated EB standard absorbance curves. Data were expressed as μg of EB/g of wet weight tissue.

#### Angpt-2 mRNA expression measurement

Separately from the above mentioned two experiments of the plasma Angpt-2 concentration measurement and vascular permeability measurement, mRNA expression in liver was measured at 1, 2, and 6 h after antigen injection. The reason why the liver was chosen is that the present rat anaphylaxis model showed strong local anaphylaxis reactions in liver [18, 19]. At each time point, four rats instrumented similarly to the plasma Angpt-2 concentration experiment were used in both the shock and control groups. Total RNA was extracted from the liver by using the RNeasy mini kit (Qiagen, Venlo, The Netherlands) and converted to complementary DNA with ReverTra Ace quantitative PCR (qPCR) Real-Time Master Mix (Toyobo, Osaka, Japan). The expression levels were determined by a QuantStudio 12K Flex Real-Time PCR System (Thermo Fisher Scientific, Waltham, USA) with TaqMan probe for angiopoietin-2 (Angpt2, Rn01756774_m1) and TaqMan master mix (Thermo Fisher Scientific). Angpt-2 mRNA level was normalized with Glyceraldehyde-3-phosphate dehydrogenase (Gapdh, Rn01775763_g1) mRNA.

#### Plasma TNF-α concentration measurement

In the Angpt-2 mRNA expression experiments, we also measured plasma levels of TNF-α. Blood samplings were performed only two times of the initial time and the endpoint of 1, 2, or 6 h after antigen to avoid multiple samplings. Plasma TNF-α levels were measured using the Rat TNF-α Quantikine ELISA Kit (R&D Systems) according to manufacturer’s guidelines and assay procedures are the same as for the measurement of plasma Angpt-2 levels.

#### Tissue water content measurement

In the Angpt-2 mRNA expression experiments, we also measured tissue water content at 6 h after antigen injection. Tissue water content was measured using the wet/dry method to examine the possibility that EB measurement of the organ with edema could cause underestimation of vascular permeability. The tissues of the organs such as trachea, bronchus, mesentery, intestine and skeletal muscle were removed at 6 h after antigen injection. The tissues in preweighed plastic tubes were weighed at the end of the experiment and weighed again after drying at 100°C for 24 h in a drying oven to evaporate all water content. The wet-to-dry ratio was calculated using the following formula: (wet weight-dry weight)/wet weight.

### Anesthetized rat experiment

Ovalbumin-sensitized (n = 7) and non-sensitized (n = 7) rats were anesthetized with an intraperitoneal injection of urethane (1.2 g/kg), and placed supinely on a heating pad [20]. The left femoral artery and vein were catheterized in a manner similar to the unanesthetized rat experiment for measurement of MBP and injections of the antigen. At baseline, and at 3, 10, 30, and 60 min after antigen injection, blood (0.3 ml) was sequentially sampled for measurements of plasma Angpt-2 concentrations and Hct, as described above.

### Statistics

Results are expressed as means ± SD, unless otherwise mentioned. For the statistical analysis of the values of measurement, intragroup and between-group comparisons were performed using one-way and two-way analysis of variance for repeated measures. When a significant difference was observed with the two-way analysis of variance, paired comparisons were made within a group and between groups by using the Dunnet and Tuckey posttest, respectively. Significance was assumed when P value was less than 0.05 (two tailed). All statistical analyses were performed using KaleidaGraph 4.0 (SAS Institute Inc., Cary, NC, USA).

## Results

### Unanesthetized rat experiment

#### Blood pressure and hematocrit

**Fig 1:**
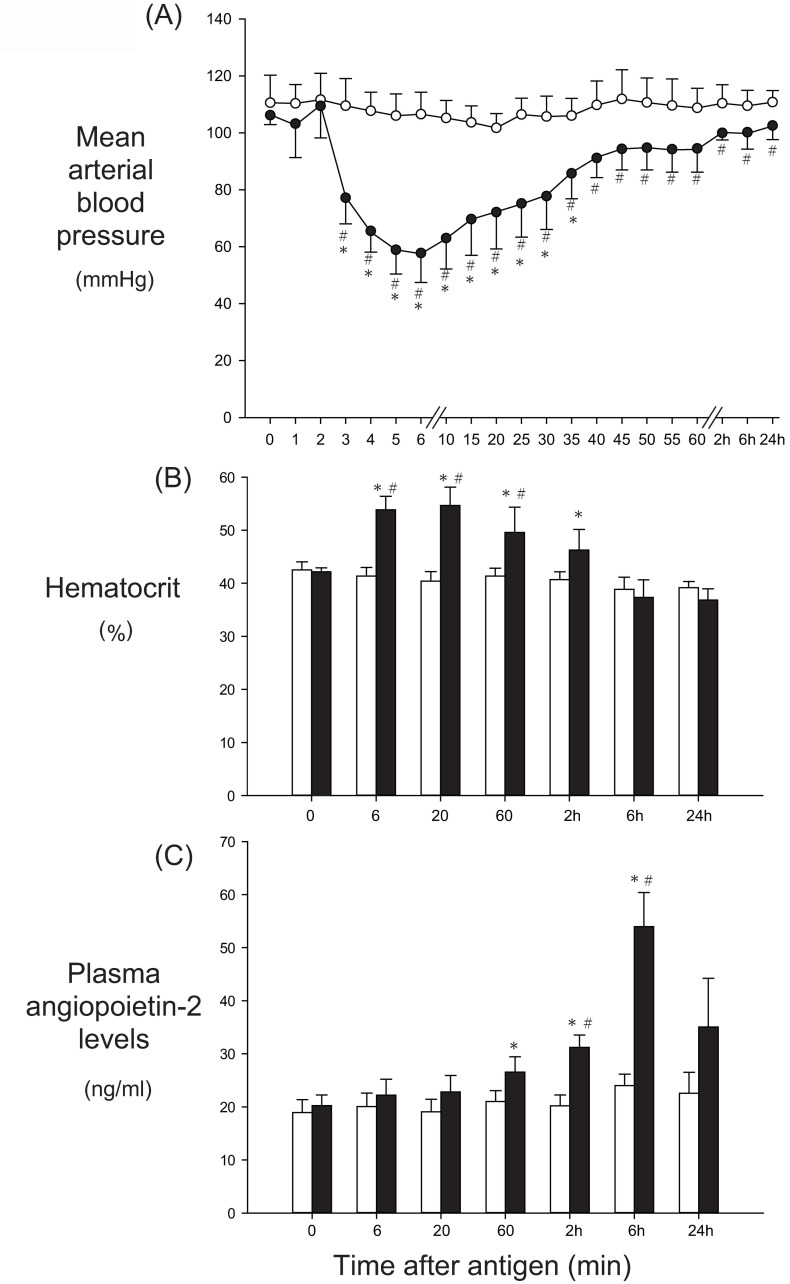
The effects of anaphylaxis on the time course changes in the mean arterial blood pressure (A), hematocrit (B) and plasma concentrations of angiopoietin-2 (C) in unanesthetized rats. In Fig 1A, open circle, the control group (n = 6); closed circle, the shock group (n = 6): Values are means ± SD; *P<0.05 vs. baseline; #P<0.05 vs. the control group. In Fig 1B and 1C, white bars, the control group; black bars, the shock group: Values are means ± SD; *P<0.05 vs. baseline; #P<0.05 vs. the control group.

In the shock group, transient systemic hypotension occurred after antigen injection, as shown in Fig 1A: MBP decreased from the baseline of 106±3 mmHg to the nadir of 58±10 mmHg at 6 min, followed by recovery to 94±7 at 45 min. The Hct increased significantly from the baseline of 42±1% to 54±3% and 55±4% at 6 and 20 min after antigen, respectively, and thereafter began to decrease, reaching 46±4% at 2 h, comparable to the baseline (Fig 1B). In the control group, MBP and Hct did not change significantly from the baseline throughout the experimental period (Fig 1A and 1B).

#### Plasma Angpt-2 concentrations

After antigen injection, plasma Angpt-2 levels in the shock group did not change significantly from the baseline of 20±2 ng/ml until 1 h (27±3 ng/ml; 135% baseline), as shown in Fig 1C, and thereafter they reached the peak level of 54±6 ng/ml, 2.7-fold baseline, at 6 h, followed by a return to the baseline levels at 24 h. In the control group, plasma Angpt-2 levels did not change significantly from the baseline of 19±2 ng/ml throughout the experimental period.

#### Plasma cytokine concentrations

**Fig 2:**
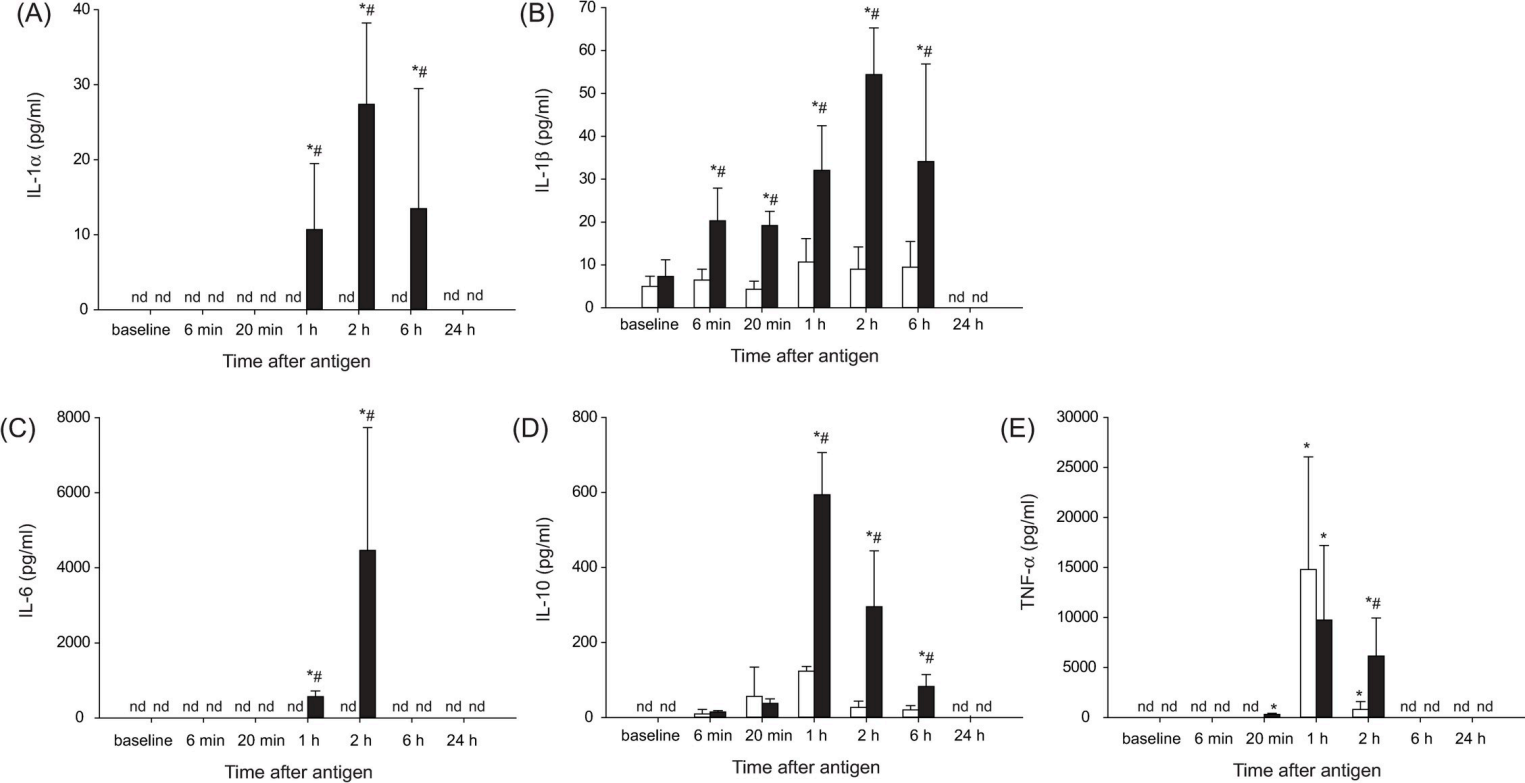
Plasma concentrations of IL-1α (A), IL-1β (B), IL-6 (C), IL-10 (D), and TNF-α (E) after antigen injection in the shock (n = 6, black bars) and control (n = 5, white bars) group. Values are means ± SD; *P<0.05 vs. baseline; #P<0.05 vs. the control group; nd, not significantly detected.

To try to determine the triggers to have elevated plasma Angpt-2 levels at 1–6 h after antigen, cytokine levels were measured. IL-2, IL-4, IL-5, IL-12, IL-13, IFN-γ, or GM-CSF was not detected in either the shock or control group throughout the experimental period. In contrast, IL-1α, IL-1β, IL-6, IL-10, and TNF-α were significantly detected in the shock group at 1 or 2 h, but not 24 h after antigen, as shown in Fig 2. Of note, plasma levels of these significantly detected cytokines peaked at 1 or 2 h, but not at 6 h after antigen, when plasma Angpt-2 reached the peak.

#### Vascular permeability

**Fig 3:**
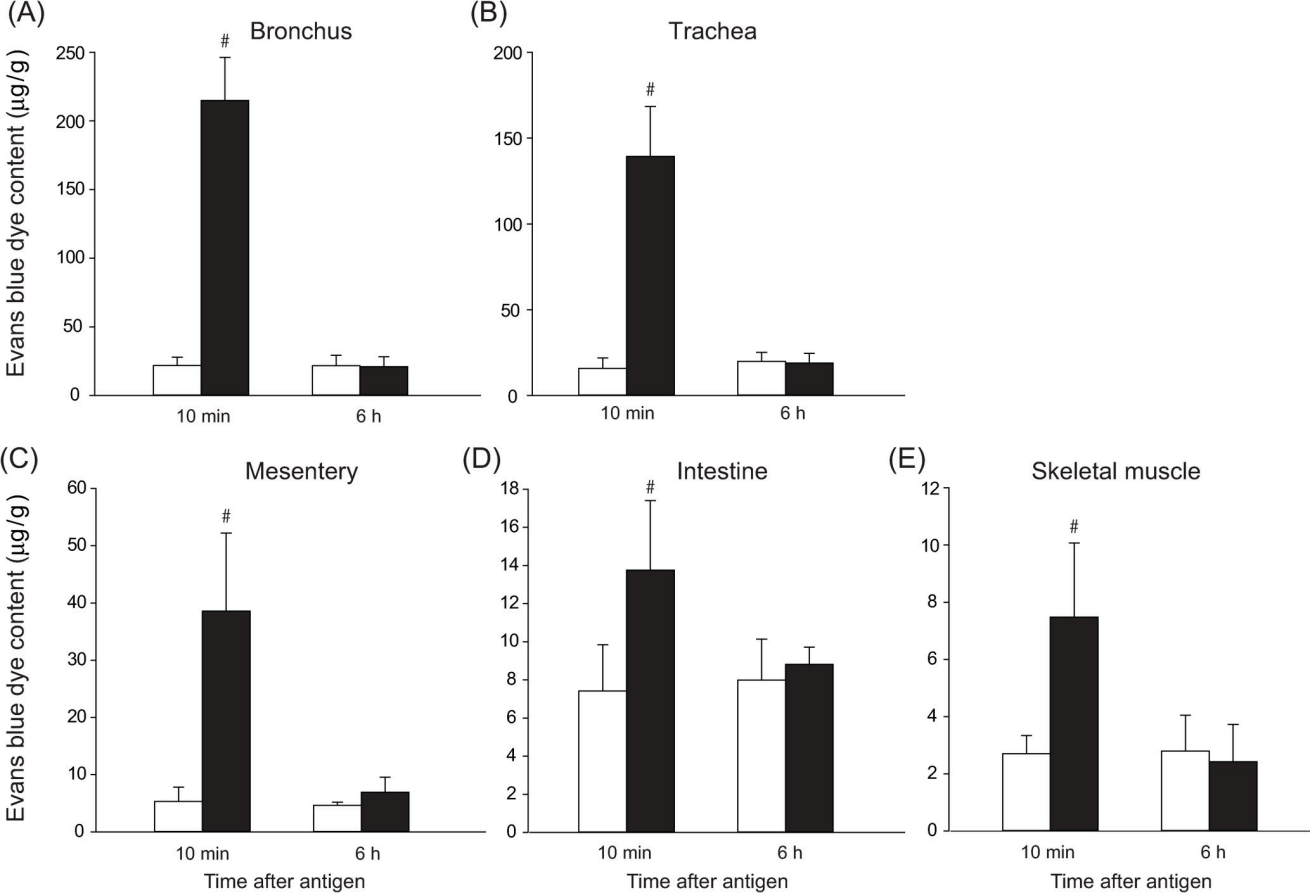
The vascular permeability of the bronchus (A), trachea (B), mesentery (C), intestine (D) and skeletal muscle (E), as evaluated by Evans blue dye contents, at 10 min and 6 h after antigen injection in the shock (n = 5, black bars) and control (n = 5, white bars) group. Values are means ± SD; #P<0.05 vs. the control group.

As shown in Fig 3, EB contents at 10 min after antigen in the shock group were significantly greater by 778% for the trachea, 876% for the bronchus, 623% for the mesentery, 85% for the intestines and 177% for the skeletal muscle than those in the control group. Of note, the EB levels at 6 h in the shock group were not significantly different from those in the control group. Table 1 shows the results of MBP in the vascular permeability study. MBP changes during the vascular permeability study in the shock and control groups were similar to those when Angpt-2 levels were measured, as described above.

**Table 1:** Mean arterial blood pressure (mmHg) in the vascular permeability study.

	baseline	6 min	10 min	1 h	6 h
control 10 min (n = 5)	115±7	117±6	117±7		
shock 10 min (n = 5)	124±7	72±8*^#^	68±9*^#^		
control 6 h (n = 5)	119±9	117±9	115±9	113±8	114±2
shock 6 h (n = 5)	122±7	70±13*^#^	64±9*^#^	102±9	111±6

Means±SD

*p<0.05 vs. baseline

#p<0.05 vs. control group.

#### Angpt-2 mRNA expression

**Fig 4:**
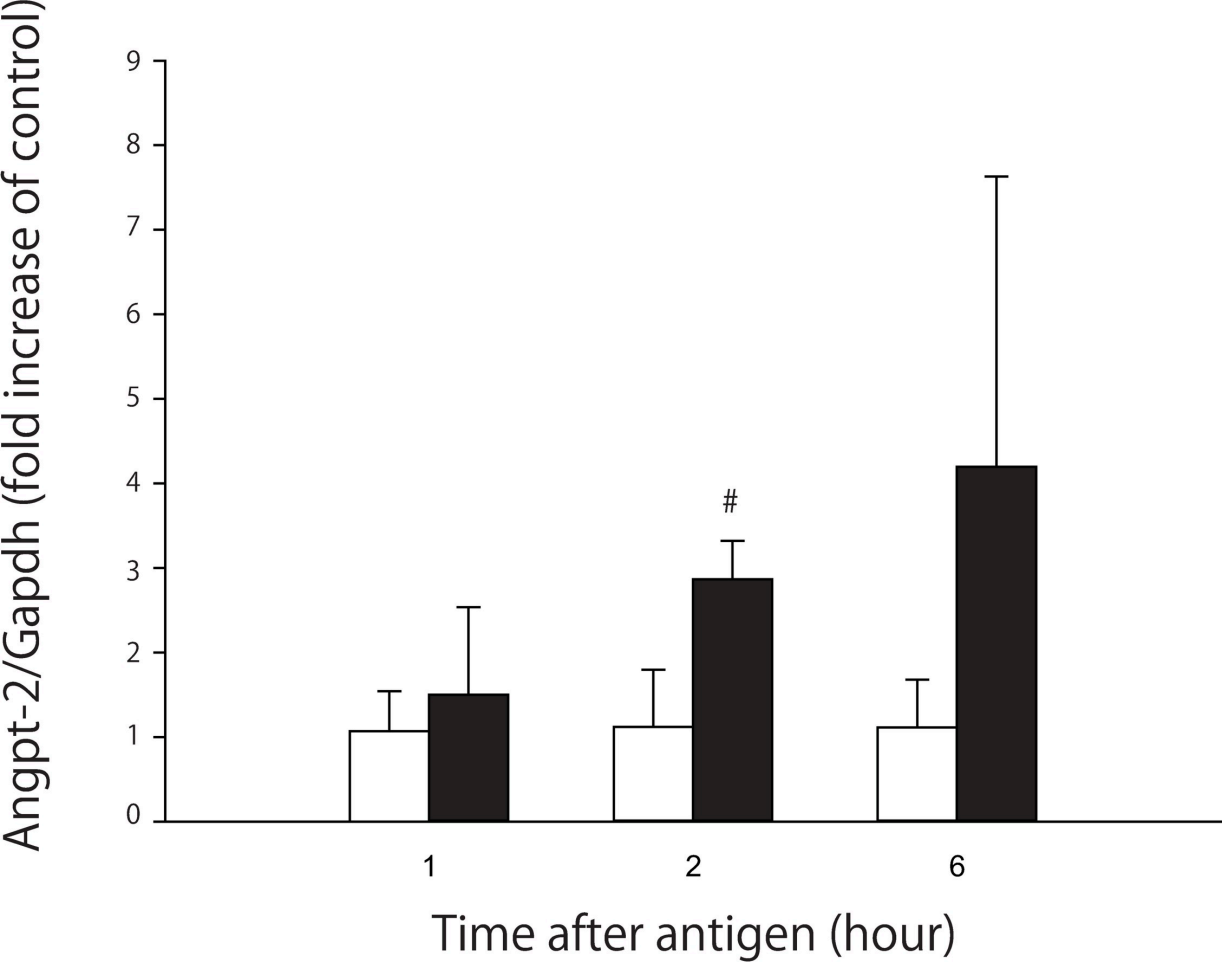
Angpt-2 mRNA levels in the liver at 1, 2 and 6 h after antigen injection in the shock (black bars) and control (white bars) group. The sample numbers are four for each time point and group. Values are means ± SD; #P<0.05 vs. the control group.

To examine whether the increase in plasma Angpt-2 levels at 6 h is due to increased gene transcription, we measured Angpt-2 mRNA levels in the liver at 1, 2 and 6 h after antigen injection. In the shock group, Angpt-2 mRNA increased significantly 3-fold at 2 h after antigen, and it also tended to increase, but not significantly, at 6 h (Fig 4). Table 2 shows MBP changes during the Angpt-2 mRNA study in the shock and control groups: MBP changed similarly to that when plasma Angpt-2 levels were measured, as described above.

**Table 2:** Mean arterial blood pressure (mmHg) in the Angpt-2 mRNA study.

	baseline	6 min	1 h	2 h	6 h
control 1 h (n = 4)	115±6	112±10	116±9		
shock 1 h (n = 4)	120±5	65±11*^#^	117±6		
control 2 h (n = 4)	116±3	118±4	121±5	117±4	
shock 2 h (n = 4)	116±7	68±5*^#^	113±3	116±5	
control 6 h (n = 4)	118±10	118±9	116±6	nm	120±4
shock 6 h (n = 4)	115±6	63±17*^#^	114±11	nm	109±6

Means±SD

*p<0.05 vs. baseline

#p<0.05 vs. control group; nm, not measured.

#### Plasma TNF-α concentrations

**Fig 5:**
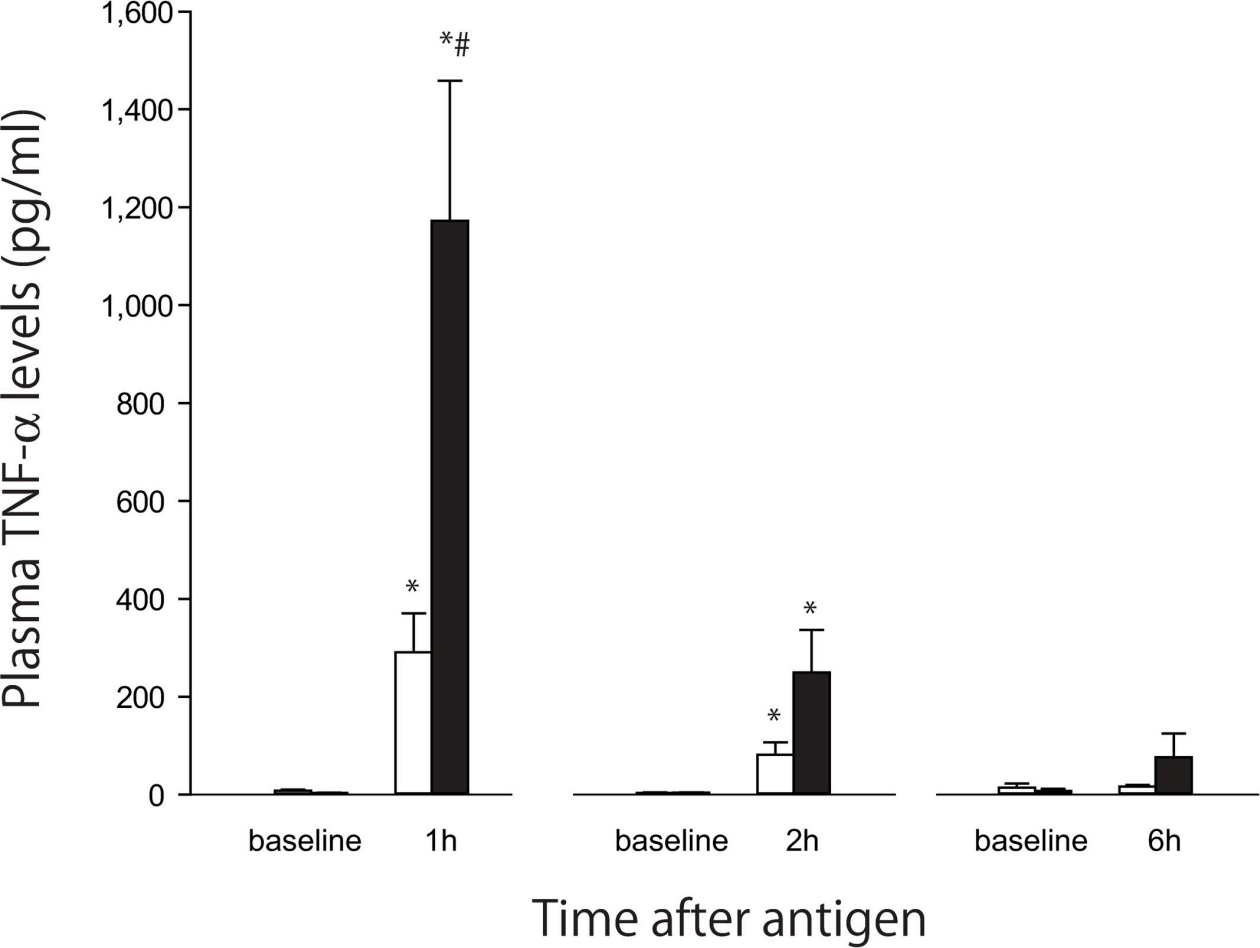
Plasma TNF-α levels at 1, 2 and 6 h after antigen injection in the shock (black bars) and control (white bars) group. The sample numbers are four for each time point and group. Values are means ± SE; *P<0.05 vs. baseline; #P<0.05 vs. the control group.

In the present study, we unexpectedly found that plasma TNF-α levels increased at 1 h time point in the control group, as shown in Fig 2E. We hypothesized that this TNF-α increase is caused by the procedures of multiple arterial blood samplings because the baseline TNF-α levels were not increased but undetectable (Fig 2E). To examine this assumption, in this additional experiments, serial blood samplings were not performed but only one time sampling at the endpoint at 1, 2 or 6 h after antigen injection. Consistent with this assumption, as shown in Fig 5, although plasma TNF-α levels of the control group increased at 1 h after antigen, they were much smaller than not only those of previous experiments (Fig 2E) but also the shock group (Fig 5). In the shock group, plasma TNF-α levels increased to 1,172±285 (SE) pg/ml at 1 h after antigen, with decline at 2 h and returned to the levels not significantly higher than the baseline at 6 h.

#### Tissue water content

**Fig 6:**
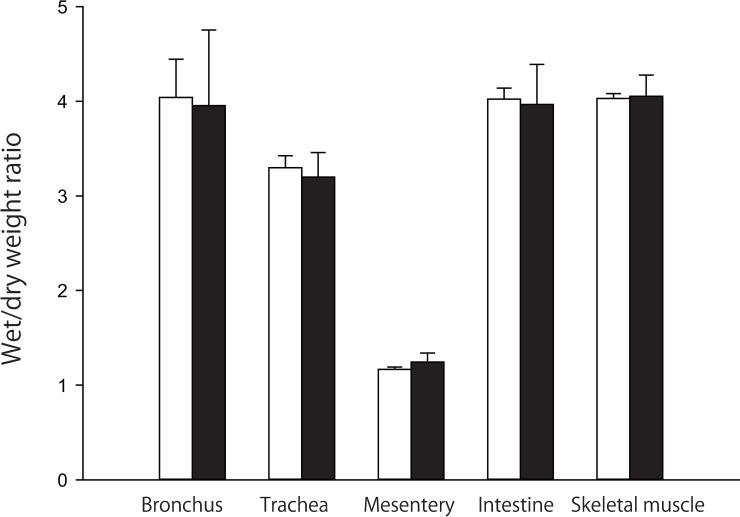
Wet/dry weight ratios of various tissues at 6 h after antigen injection in the shock (n = 4, black bars) and control (n = 4, white bars) group. Values are means ± SD.

To examine whether the wet weights of the organs studied in the EB experiment were greater in the shock group than in the control group, we measured the wet/dry weight ratios of the organs at 6 h after antigen in the Angpt-2 mRNA experiments. The wet/dry weight ratios in the shock group were almost the same as those in the control group, as shown in Fig 6, indicating that the organs in the shock group were not edematous.

### Anesthetized rat experiment

**Fig 7:**
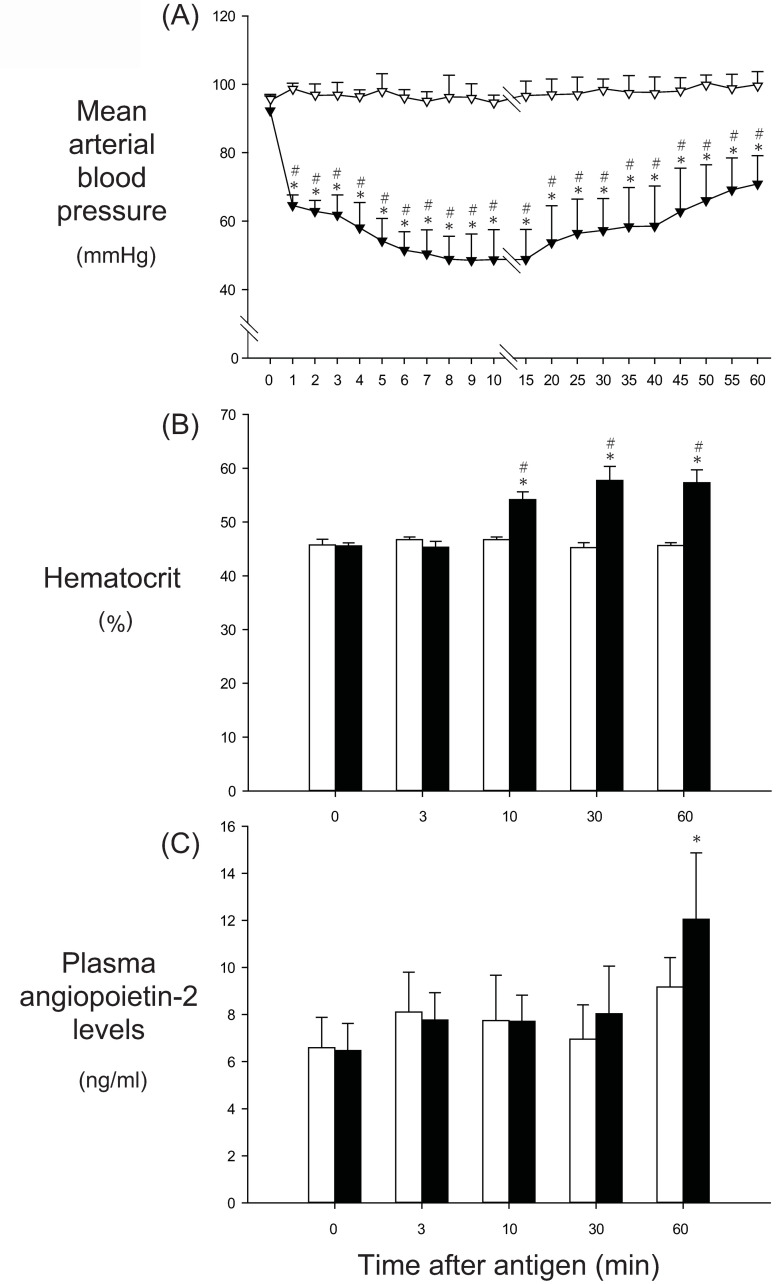
The effects of anaphylaxis on the time course changes in the mean arterial blood pressure (A), hematocrit (B) and plasma concentrations of Angpt-2 (C) in anesthetized rats. In Fig 7A, open triangle, the control group (n = 7); closed triangle, the shock group (n = 7): Values are means ± SD; *P<0.05 vs. baseline; #P<0.05 vs. the control group. In Fig 7B and 7C, white bars, the control group; black bars, the shock group: Values are means ± SD; *P<0.05 vs. baseline; #P<0.05 vs. the control group.

In the shock group, transient systemic hypotension occurred after antigen injection, as shown in Fig 7A: MBP decreased from the baseline of 92±4 mmHg to the nadir of 49±8 mmHg at 9 min, followed by partial recovery to 71±8 at 60 min. The Hct increased significantly from the baseline of 46±1% to the peak of 58±3% at 30 min after antigen (Fig 7B). After antigen injection, plasma Angpt-2 levels in the shock group did not change significantly from the baseline of 6.5±1.2 ng/ml until 1 h (12±2.8 ng/ml; 185% baseline), as shown in Fig 7C. In the control group, MBP, Hct or plasma Angpt-2 levels did not change significantly throughout the experimental period (Fig 7).

## Discussion

In the present study, we found that plasma Angpt-2 levels did not significantly increase until 60 min after antigen injection, although increased vascular permeability was observed at 10 min and plasma extravasation, as evaluated by increased Hct, peaked at 20 min. Plasma Angpt-2 levels reached the peak at 6 h, when vascular permeability was not increased and plasma extravasation was subsided. This is the first study to determine the plasma Angpt-2 levels during systemic anaphylaxis in in vivo animals.

Angpt-1 and Angpt-2 are secreted endothelial growth factors that bind to Tie-2 receptors to regulate endothelial permeability [21] and the actions of these two Angpt subtypes oppose each other. Angpt-1 released primarily from pericytes protects against vascular leak by constitutively maintaining endothelial junctions [22], whereas Angpt-2 stored in Weibel-Palade bodies of endothelial cells increases vascular permeability by competing with Angpt-1 for binding to Tie-2 [23]. Actually, Angpt-2 is reported to contribute to increased vascular permeability in clinical disorders such as sepsis [7, 24] and acute lung injury [8, 25, 26], and their experimental animal models [27, 28]. We here for the first time showed that plasma Angpt-2 levels were increased during systemic anaphylaxis, the hallmark of which is increased vascular permeability in unanesthetized and anesthetized rats.

The rationale for examining whether Angpt-2 is released during systemic anaphylaxis was based on the following lines of evidence: Angpt-2 is stored in the Weibel-Palade body along with von Willebrand factor and P-selectin in the endothelial cells, and is released via degranulation of that body in response to a large number of secretagogues [29], which include well-known anaphylactic mediators such as histamine [30], peptido-leukotrienes [10] and serotonin [11]. Epinephrine and vasopressin, the plasma levels of which are increased during systemic anaphylaxis [15, 16, 31], also cause exocytosis of the Weibel-Palade body from the endothelial cells [32, 33].

The reasons remain unknown why we could not find an increase in plasma Angpt-2 levels at an early stage within 20 min after antigen, during which increased vascular permeability and systemic hypotension occurred. We think that the above-mentioned Angpt-2 releasing secretagogues might have not been generated quantitatively enough to increase Angpt-2 to the levels detectable by ELISA in the rat anaphylaxis model of the present study. Another more plausible explanation may be related to NO, which can inhibit exocytosis of the Weibel-Palade body [34] and actually release of Angpt-2 [35]. It is well established that NO is generated and plays a crucial role in systemic anaphylaxis [5, 6]. In the present study, NO might counteract the Angpt-2 releasing action of the above mentioned secretagogues of histamine et al., resulting in attenuation of Angpt-2 release at an early stage of anaphylaxis. By the way, although potentially undetectable by ELISA, Weibel-Palade body exocytosis and Angpt-2 release 30 min after inflammatory lipopolysaccharide stimulus have been reported using immunohistochemistry in the murine vasculature [36, 37]. Thus, it is possible that we failed to detect a systemic increase in Angpt-2, although locally Angpt-2 is being secreted in an autocrine manner.

The exact mechanism for the increase of plasma Angpt-2 levels, which started to elevate at 1 h and reached the peak at 6 h, after antigen is not known. We speculated that the increased plasma concentrations of several cytokines such as IL-6 and TNF-α, which peaked at 1 or 2 h, may cause the early increase of plasma Angpt-2 [9, 13, 14] and that the subsequent increase in plasma Angpt-2 levels at 6 h may be at least in part due to increased gene transcription: Angpt-2 mRNA expression in the liver increased at 2 h and showed an increasing tendency at 6 h (Fig 4).

The physiological significance of the increased Angpt-2 during anaphylaxis is not known. The peak plasma Angpt-2 level was observed at 6 h after antigen. However, at that time, Hct decreased to the baseline values, indicating that plasma extravasation was recovering. Actually, vascular permeability evaluated with EB was not increased, but recovered to the same level as in the control, as shown in Fig 3. These findings suggest that elevated Angpt-2 did not increase vascular permeability. We assume that the plasma levels of Angpt-2 were too low to produce the permeability increasing action. Supporting this notion, in the present study, the maximum level of Angpt-2 at 6 h after antigen was nearly 50 ng/ml, which was the minimal [38] or lower [39] level to activate Tie-2 receptors and to evoke subsequent intracellular signal transduction for exerting Angpt-2 actions. A more plausible explanation is based on well-established notion that the antagonistic activity against Angpt-1 exhibited by Angpt-2, the property to increase vascular permeability, is induced in inflammation [12, 40]. There is a possibility that in the present anaphylaxis model, inflammation is rapidly settled down, resulting in inhibition of Angpt-2 antagonistic activity, and thus, less permeability increase.

There are limitations in the present study. First, the baseline plasma Angpt-2 values of 20 ng/ml in the present awake rats are much higher than the values in human plasma samples. In additional experiments of the anesthetized rats, we found that the baseline Angpt-2 levels were approximately 6 ng/ml. The exact reasons why the baseline Angpt-2 levels of the awake rats were 3-fold higher than those of the anesthetized rats were not known. We assume that the invasive procedures of surgery done in awake rats, which are composed of making a subcutaneous tunnel to pass the catheter tubings between the groin and neck, and the placement of the catheters and their connecting tubings for 24 h, might have caused inflammation, resulting in the rise of plasma Angt-2 levels. Nonetheless, the plasma Angpt-2 values at 1 h after antigen injection were similarly increased from the baseline values in anesthetized rats and awake rats, as shown in Figs 7 and 1. We believe that the antigen-induced increase in plasma Angpt-2 levels is substantial in the present awake rats.

Secondly, we measured the EB contents of wet tissues, but not that of dry ones. If edema occurred, EB contents in the wet tissue should be underestimated. In the present study we found that EB contents increased at 10 min, but not at 6 h after antigen. Thus, there is a possibility that EB values of 6 h specimens might be underestimated. However, in the separate experiments, we measured the wet-dry ratio of the organs such as trachea, bronchus, mesentery, intestine and skeletal muscle at 6 h after antigen injection. There were no significant differences in the wet-dry ratio between the shock and control groups, as shown in Fig 6. These results suggest that there was little or no edema in the organs of the shock group at 6 h. This finding is also consistent with no significant difference in Hct at 6 h between the shock and control groups (Fig 1B). Taken together, the EB values at 6 h seem not to be underestimated, indicating that the vascular permeability was not increased at 6 h after antigen.

Finally, the plasma TNF-α level measured in the control group of the initial study (Fig 2E) was as high as that in the shock group. Because the baseline TNF-α was not increased, the reason for the increase at 1 h should be ascribable to the invasive procedures during experiments. The possible procedures include the initial flushing of the arterial and venous lines for MBP recording and injections of the antigen, respectively, and multiple arterial samplings. In additional experiments, even though serial blood samplings were not performed but only one-time sampling was performed at the endpoint time of 1, 2 or 6 h after antigen injection, we again found a small but significant increase in plasma TNF-α values at 1 h after antigen injection in the control rats, as shown in Fig 5. Thus, the initial flushing rather than the multiple arterial blood samplings may account for the TNF-α increase. The initial flushing of the arterial and venous lines might have caused possibly microembolism, resulting in inflammation.

## Conclusions

Plasma Angpt-2 levels began to increase at 1 h reaching the peak at 6 h with a return to the baseline at 24 h after antigen injection in unanesthetized rats. In anesthetized rats, plasma Angpt-2 levels also increased at 1 h after antigen. Plasma extravasation and increased vascular permeability occurred within 10 min after antigen, while they subsided at 6 h. Plasma levels of cytokines such as IL-1α, IL-1β, IL-6, IL-10, and TNF-α peaked 1 or 2 h after antigen. Angpt-2 mRNA increased at 2 h and showed an increasing tendency at 6 h. Thus, we conclude that the plasma Angpt-2 level is elevated presumably due to increased cytokines and enhanced gene transcription during anaphylaxis in anesthetized and unanesthetized rats.
